# Recent Advances in the Treatment of Spasticity: Extracorporeal Shock Wave Therapy

**DOI:** 10.3390/jcm10204723

**Published:** 2021-10-14

**Authors:** En Yang, Henry L. Lew, Levent Özçakar, Chueh-Hung Wu

**Affiliations:** 1 Department of Physical Medicine and Rehabilitation, National Taiwan University Hospital, Taipei 100, Taiwan; youngforever0826@gamil.com; 2 Department of Physical Medicine and Rehabilitation, Virginia Commonwealth University School of Medicine, Richmond, VA 23298, USA; henrylew@hawaii.edu; 3 Department of Communication Sciences and Disorders, John A Burns School of Medicine, University of Hawaii, Honolulu, HI 96822, USA; 4 Department of Physical and Rehabilitation Medicine, Hacettepe University Medical School, Ankara 06100, Turkey; lozcakar@yahoo.com; 5 Department of Physical Medicine and Rehabilitation, National Taiwan University Hospital Hsin-Chu Branch, Hsinchu 300, Taiwan; 6 Department of Physical Medicine and Rehabilitation, College of Medicine, National Taiwan University, Taipei 100, Taiwan

**Keywords:** extracorporeal shock wave therapy, muscle spasticity, rehabilitation, review

## Abstract

Spasticity is a common sequala of the upper motor neuron lesions. For instance, it often occurs in the first 4 weeks after stroke and is seen in more than one-third of stroke survivors after 12 months. In recent years, extracorporeal shock wave therapy (ESWT) has been recognized as a safe and effective method for reducing muscle spasticity. Possible/relevant mechanisms include nitric oxide production, motor neuron excitability reduction, induction of neuromuscular transmission dysfunction, and direct effects on rheological properties. There are two types of ESWT, focused and radial, with the radial type more commonly applied for treating muscle spasticity. Concerning the optimal location for applying ESWT, the belly muscles and myotendinous junction seem to produce comparable results. The effects of ESWT on spasticity are known to last at least four to six weeks, while some studies report durations of up to 12 weeks. In this review, the authors will focus on the current evidence regarding the effectiveness of ESWT in spasticity, as well as certain technical parameters of ESWT, e.g., the intensity, frequency, location, and number of sessions. The pertinent literature has been reviewed, with an emphasis on post-stroke upper limbs, post-stroke lower limbs, cerebral palsy, and multiple sclerosis. In short, while ESWT has positive effects on parameters such as the modified Ashworth scale, mixed results have been reported regarding functional recovery. Of note, as botulinum toxin injection is one of the most popular and effective pharmacological methods for treating spasticity, studies comparing the effects of ESWT and botulinum toxin injections, and studies reporting the results of their combination, are also reviewed in this paper.

## 1. Introduction

Spasticity is a neurological manifestation caused by upper motor neuron (UMN) syndrome. It has been defined as a velocity dependent increase in muscle tone caused by the increased excitability of muscle spindles [1].

Spasticity is one of the most common sequalae in stroke patients. It affects 43% of stroke patients 12 months after onset [2]. In addition, spasticity can often be detected in the first 4 weeks after a cerebral vascular event [3]. Involvement of the upper extremities is more common than the lower extremities and is proportional to the severity of the upper-limb functional impairment [4]. In addition to stroke, multiple sclerosis (MS), cerebral palsy (CP), and neurological traumas are other disorders in which spasticity is also commonplace. Earlier studies showed that spasticity is experienced by 60–90% of persons with MS [5], 69.8% of children with CP [6], and one in six people with traumatic brain injury [7].

Various non-pharmacological interventions have been studied for the management of spasticity. Some examples include stretching and passive movements, transcutaneous electric nerve stimulation, transcranial direct current stimulation, shock waves, vibratory stimulation, electromyography biofeedback, repetitive trans-cranial magnetic stimulation, therapeutic ultrasound, acupuncture, orthoses, thermotherapy, and cryotherapy [8]. Although the range of non-pharmacological approaches is wide, there is a lack of high-quality evidence for most of the aforementioned modalities.

In recent years, increasing evidence has been collected of extracorporeal shock wave therapy (ESWT) being a safe and effective alternative for reducing muscle spasticity. Indeed, ESWT is considered a valuable adjuvant modality to standard treatment and rehabilitation [9,10]. Accordingly, this narrative review will focus on the current evidence pertaining to the etiology, duration, and outcomes of ESWT in spasticity. Clinical and methodological aspects of ESWT will also be addressed.

We used PubMed databases to search English papers. The MeSH keywords used were: “shock waves”, “extracorporeal shock wave therapy”, “muscle spasticity”, “stroke”, “cerebral palsy”, and “multiple sclerosis”.

We looked for papers discussing muscle spasticity and shock waves. Different etiologies of spasticity were found, including stroke, cerebral palsy, and multiple sclerosis. We used different inclusion criteria in different fields. For studies on poststroke spasticity, which is a well-recognized category, we included only randomized controlled studies and excluded all papers without proper grouping and randomization. In papers studying patients with cerebral palsy and multiple sclerosis, in which randomized controlled trials were relatively scarce, we included all original clinical trials, regardless of study design.

**Figure 1:**
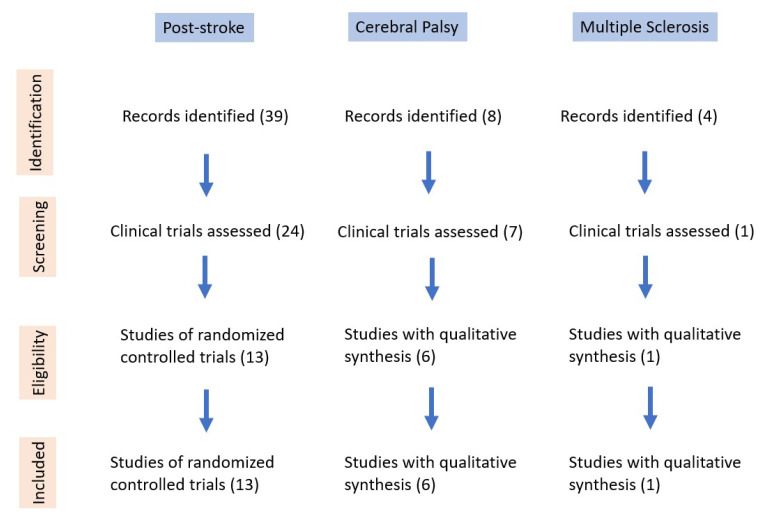
Diagram of selection process and identification of eligible studies.

Articles were reviewed by the first author (E.Y.) and checked by the co-authors (H.L., L.Ö. and C.W.). Discrepancies were resolved by discussion with consensus. Figure 1 demonstrates our selection process and the identification of eligible studies.

## 2. Effects on Neuromuscular Tissues

Shock waves are generated via rapid propagation of suddenly increased pressure in three-dimensional space, resulting in sequences of biphasic acoustic impulses with high energy. Shock waves can be focused to target specific tissues, without affecting the overall structure [11].

**Figure 2:**
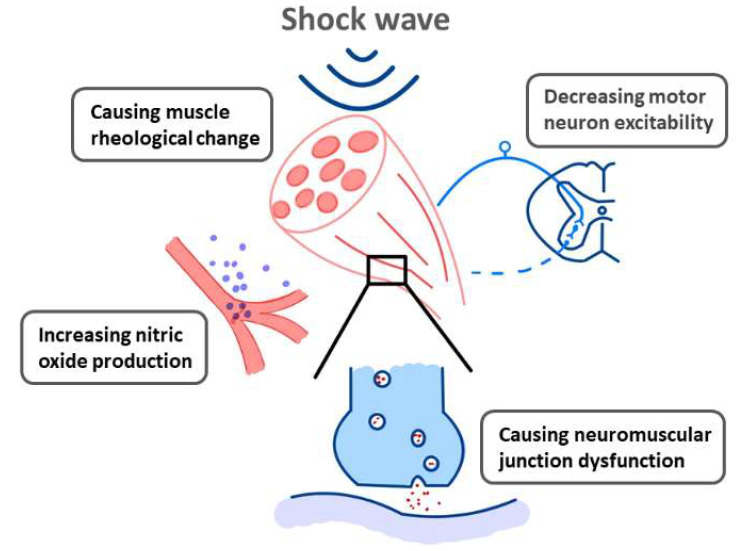
Proposed mechanisms of shock wave effects in spasticity, according to Xiang et al. [14], Moon et al. [15], Kenmoku et al. [16], and Manganotti et al. [17].

In clinical practice, ESWT has been widely used to treat musculoskeletal diseases, such as pain, inflammation, and ligament injury. It is believed to exert mechanical effects and to induce changes in tissue physiological response. While the former includes tissue regeneration, neovascularization, and resorption of calcium deposition [12], the latter consists of changes in epithelial cell permeability and the formation of free radicals, nitric oxide (NO), and variable growth factors [13]. Although the mechanism(s) behind the effects of ESWT in spasticity remain uncertain, pertinent studies have suggested the following possibilities: (Figure 2).

### 2.1. Inducing NO Production

Increasing NO synthesis, which is necessary in neuromuscular junction formation in the peripheral nervous system, is the most well-known mechanism of ESWT. NO can further increase muscle and tendon neovascularization, thereby improving muscle stiffness [14]. In addition, NO also acts on the central nervous system, affecting certain physiological functions (e.g., neurotransmission and synaptic plasticity) [18].

### 2.2. Reducing Motor Neuron Excitability

The literature suggested that ESWT can reduce the hyperexcitability of the alpha motor neuron [19]. According to Leone et al. [20], motor neuron excitability can be reduced by tendon pressure [15].

However, more recent studies with electrophysiological measures did not report a significant difference in spastic muscle after ESWT treatment [21,22], suggesting that neuronal effects may not be the primarily mechanism of ESWT on spasticity [21].

### 2.3. Dysfunction in Neuromuscular Transmission

ESWT can reduce the number of acetylcholine receptors in the neuromuscular junction. In one study, degenerated acetylcholine receptors were found in all the muscles of Sprague–Dawley rats treated with shock waves [16]. Furthermore, electrodiagnostic testing showed that compound motor action potential amplitudes were significantly lower on the treated vs. control side. The therapeutic effect was noted starting from the day of treatment until the 6th week, and no more than the 8th week. As such, the authors reported that the application of shock waves to muscle induced only a transient dysfunction of nerve conduction at the neuromuscular junction [16].

### 2.4. Affecting Rheological Properties

The mechanism of ESWT made a neural and peripheral contribution to muscle spasticity [21]. A peripheral effect, also known as a non-neural effect, was recognized through its effects on the rheological properties and fibrosis of chronic hypertonic muscles. Considering the therapeutic effects of ESWT on bones and tendons, Manganotti et al. [17] proposed that a reduction of spasticity could be achieved by improving the stiffness of connective tissues by directly acting on fibrosis in the spastic muscles. Dymarek et al. [23] used infrared thermal imaging (IRT) to measure trophic conditions in spastic muscles after rESWT treatment. They found a significant increase in IRT values after ESWT, suggesting an improvement of the trophic conditions of the spastic muscles. Leng et al. [21] used the NeuroFlexor method, a myotonometer, and electrical impedance myography, and found a significant decrease in muscle tone, stiffness, and viscosity after ESWT. They proposed that ESWT could cause a biological response that alternates between metabolic and proliferative processes, affecting the muscle fibrosis and rheological properties.

## 3. Radial vs. Focused ESWT

There are two main types of generators that can produce shock waves: focused ESWT (fESWT), and radial ESWT (rESWT). These two types differ, not only in their physical properties and mode of generation, but also in the magnitude of the standard parameters used and the penetration depths achieved [24].

fESWT is generated by electromagnetic, electrohydraulic, and piezoelectric sources. The pressure in fESWT increases rapidly, and the energy can be absorbed as deep as 12 cm [11]. As the dispensed energy is relatively low, damage to the skin and the underlying soft tissues is limited. rESWT is generated by means of a pneumatic system. The maximum energy is at the probe tip and transduced radially into the tissue [25]. The pressure increases much more slowly and the depth of energy absorbency is also very shallow, i.e., only 3–4 cm deep [11].

Overall, fESWT is more intense within a targeted area, while rESWT has a more widespread but superficial region of action [11]; therefore, rESWT is considered a less invasive tool and is more appropriate for physiotherapy purposes. [22] Nonetheless, there is no conclusive evidence regarding which type of ESWT is more effective in treating spasticity [11]. 

## 4. Site and Duration of Application

The effects of ESWT are achieved through the penetration of energy into a specific region; therefore, it is crucial to know the exact tissue targeted. However, only a limited number of reports have addressed the issue of the most optimal location for applying ESWT to muscles [26].

According to the hypothesis that ESWT reduces motor neuron excitability, shock waves should be administered at myotendinous junctions, where the Golgi tendon organ resides. Based on the theory that the effects of ESWT are due to the disruption of neuromuscular transmission and direct changes in the rheological properties, the belly muscles seem to be a preferred site for applying shock waves. To answer this question, Yoon et al. [26] conducted a study in which patients were divided into a control and two ESWT groups (i.e., targeting the belly muscles and the myotendinous junction). The study results showed that the MAS and MTS evaluations improved after the treatment, whereas the two ESWT groups were not different.

As for muscle selection, Li et al. [27] investigated whether the agonist or the antagonist muscles should be treated. They performed a study recruiting post-stroke patients with spasticity. Patients were randomly divided into three groups: control, rESWT on agonist muscles, and rESWT on antagonist muscles. rESWT had an effect on reducing MAS score and VAS score in both agonist and antagonist groups. However, there was no effect on active functions or the swelling of upper limbs. According to Li et al. [27], a possible mechanism of reducing spasticity by treating the antagonist muscles is through pain relief, including improving complex regional-pain syndromes.

The effects of ESWT on spasticity are known to last at least four to six weeks in patients with stroke or CP [28,29]. Manganotti and Amelio [17] further studied the long term effects of ESWT and reported that a reduction in pain and MAS grades, as well as improved motor function, remained at 12 weeks [30].

## 5. Adverse Effect

Shock waves are generally a safe modality, but patients with bleeding disorders and pregnancy are still considered contraindicated to ESWT [31]. As for the side effects of ESWT, most studies revealed no obvious complications, and many studies did not report information about adverse events. Dymarek et al. [11] mentioned only 11 cases of unexpected ESWT-related side effects in their literature review. The pertinent side effects included pain (*n* = 5), lower limb muscular weakness (*n* = 2), petechiae (*n* = 3), and small bullae (*n* = 1) [32,33], all of which were well-tolerated and resolved within days.

## 6. Effect of ESWT in Different Clinical Conditions

### 6.1. Post-Stroke Upper Limb Spasticity

In this review, eight studies evaluating the effects of ESWT in post-stroke upper limb spasticity were included. All of the studies were randomized controlled trials (RCTs) with a high level of evidence. The study designs and outcome parameters are listed in Table 1, and the treatment protocols and their effects will be addressed in this section.

#### 6.1.1. Intensity, Frequency, and Dosage

The treatment protocols of each study are given in Table 2. Generally, pulse numbers were set to 1000 to 2000 for each forearm muscle. The frequency was kept between 4 to 5 Hz (Li et al. [27] used 18 Hz). A pressure of 1.5 bars was applied in most studies (Li et al. [36] and Wu et al. [37] applied 3 to 3.5 bars). The total energy flux density varied between the studies; while most of them used a relatively low energy (0.03 mJ/mm^2^), Santamato et al. [34], Yoon et al. [26], and Li et al. [27] applied greater energy (0.06–0.1 mJ/mm^2^) settings.

#### 6.1.2. Clinical Assessment

For assessing spasticity, MAS score is the most widely used method. For statistical purposes, most studies considered a MAS score of ‘1′ as 1, and a MAS score ‘1+’ as 2, and so on until 5; although Dymarek et al. [35] regarded MAS scores of ‘1+’ as a 1.5 value. Among all studies, significant MAS changes were noted after ESWT in the upper limbs of post-stroke patients. According to Li et al. [27], the response rate, defined by at least one grade of MAS improvement, was 63.3–70.4% immediately after ESWT and 66.7–74.1% after four weeks follow-up. Comparing different sites of the upper limbs, Dymarek et al. [35] found that MAS could decrease up to 0.2 grades (from 1.5 to 1.3) in the elbow, 0.4 grades (from 1.7 to 1.3) in the radiocarpal joint, and 0.7 grades (from 2.1 to 1.4) in the finger joints; with the most prominent effect noted immediately or one hour after ESWT. Wu et al. [37] found similar results, with an MAS improvement of 1.05 grades (from 3.4 to 2.35) in the wrist and 1.2 grades in the elbow (from 3.35 to 2.15). Recently, a study by Leng et al. [21] also reported up to 1 grade improvements of MAS scores (from 2 to 1) immediately after ESWT. Furthermore, when combined with botulinum toxin injections, Santamato et al. [34] found that a further drop in MAS scores could be observed, of up to 2.13 grades (from 3.5 to 1.37), at 15 days follow up.

#### 6.1.3. Functional Assessment

Fugl-Meyer Assessment (FMA) is the most commonly used tool for evaluating motor control. Wu et al. [37] reported a 47% increase in FMA scores (from 24.1 to 34.4) after a three-session treatment course. However, Leng et al. [21] found only a 14.5% increase in the FMA scores after a single session treatment, and this was not statically significant compared with the control group. These studies imply that the improvement of motor control can also be session dependent. Li et al. [36] further proved this concept and found that the differences in hand and wrist function scores were significantly larger after three-session vs. a single-session treatment.

#### 6.1.4. Other Assessments

Dymarek et al. [35] applied noncontact infrared imaging to the monitor trophic conditions of the spastic muscles. They showed that effects of ESWT on muscle properties could be detected. Park et al. [38] used myotonometric measurements to assess upper limb muscle tone and reported better results after ESWT. Leng et al. [21] used a NeuroFlexor, myotonometer and impedance myography methods to evaluate the effects of ESWT on muscles and joints and reported positive results immediately after ESWT.

### 6.2. Post-Stroke Lower Limb Spasticity

In this review, six RCTs are included regarding post-stroke lower limb spasticity treated with ESWT. Table 3 presents a summary of the study designs and outcome parameters. The treatment protocols and results will be addressed in this section.

#### 6.2.1. Intensity, Frequency, and Dosage

The protocols of ESWT for each study are listed in Table 4. Most of the parameters were similar to those of the upper limb studies, with pulse numbers ranging between 1500 to 2000 in each muscle. The frequency was between 4 and 5 Hz and the pressure was between 1 and 2 bars. The total energy flux density was generally higher in the lower limbs; however, the settings still varied among studies. The energy values were commonly around 0.1 mJ/mm^2^ (0.068–0.1 mJ/mm^2^) but were higher in the study by Radinmehr et al. [41] (0.34 mJ/mm^2^).

#### 6.2.2. Clinical Assessment

Similarly to the upper limb studies, MAS score was the most common tool used to evaluate lower limb spasticity. As in most of the upper limb studies, the MAS scoring system was applied for statistical purposes, with grade 1+ considered 2, and grades 2, 3, and 4 matched to 3, 4, and 5, respectively. In studies with one session protocol, the drop of MAS was reported as 0.64 grades (from 2.2 to 1.56) [40] and 1 grade (from 2 to 1) [41]. In studies with three treatment sessions, the amount of decrease were 0.54 grades (from 2.85 to 2.31) [26], 1.1 grades (from 2.6 to 1.5) [39], and 1.3 grades (from 3.1 to 1.8) [25]. Aslan et al. [42] found a 1.07 grade (from 2.47 to 1.4) decrease in MAS score after four treatment sessions.

Besides MAS, MTS and Tardieu angle were also used. Yoon et al. [26] found a 27.2% increase in the catch angle (from 52.38 to 66.62 degrees) after administering ESWT. Wu et al. [25] used the Tardieu angle, which is the difference between the arrest angle at slow speed and the catch angle at fast speed, and found a 35% improvement (from 20 to 13 degrees) after ESWT. Aslan et al. [42] also measured Tardieu score and found a 29.8% improvement of spasticity angle (from 17.8 to 12.5 degrees) after ESWT.

Pain scores were also used to assess lower limb spasticity. A decrease of 57.8% (VAS from 4.5 to 1.9) was reported by Taheri et al. [39], four weeks after ESWT.

#### 6.2.3. Functional Assessment

FMA, lower extremities functional scale (LEFS), gait speed, timed “up and go” test (TUG), 3-m walk duration, and 10-m walk tests were parameters used for evaluating lower limb functions. In this category, the results were relatively inconclusive.

Taheri et al. [39] found in that LEFS improved significantly compared to the baseline after one session of ESWT; while 3-m walk duration only showed an improvement after three sessions of ESWT.

In contrast, Wu et al. [25] measured 10-m walk test, but did not find an improvement after ESWT. Radinmehr et al. [41] used a TUG test and found patients had a small, 9.6% (from 21.9 to 19.8 s) improvement after ESWT, which was not clinically significant.

#### 6.2.4. Other Assessments

Lee et al. [40] used ultrasound to evaluate spasticity and reported that after ESWT, Achilles tendon length, muscle thickness, and pennation angle decreased, while muscle fascicle length increased over time. They also pointed out that with four weeks of follow-up, the differences in the ultrasonographic findings were greatest at the last follow-up. Aslan et al. [42] used ultrasound elastography to assess the elastic properties of plantar flexor muscles. However, there was no significant difference in elastic properties between the ESWT and control groups.

Radinmehr et al. [41] studied the electrophysiological changes of stroke patients by measuring the H/M ratio and H-reflex latency. However, those properties did not decrease over time, possibly implying that they are not sensitive enough to detect the relevant changes in spasticity after ESWT.

### 6.3. Spasticity in CP Patients

In this review, seven studies (including two RCTs) are included regarding the effects of ESWT on spasticity in CP. The designs and outcome parameters of those studies are presented in Table 5. Their treatment protocols and effects will be addressed in this section.

#### 6.3.1. Intensity, Frequency, and Dosage

The protocols of ESWT used in the studies are listed in Table 6. Pulse numbers ranged between 1500 and 2000 in each muscle, similar to the post-stroke protocols. However, the frequencies used in CP patients were higher, compared to the post-stroke studies, mostly ranging between 8 and 10 Hz. Gonkova et al. [45] and Park et el. [48] used relatively lower frequencies (4–5 Hz) however. Pressures of 1.5–2 bars were applied in most of the studies, but Wang et al. [46] used a lower value (0.6). The total energy flux density was generally low, i.e., mostly 0.03 mJ/mm^2^, but in the studies by Vidal et al. [44,49] it was higher (0.1–0.12).

#### 6.3.2. Clinical Assessment

Amelio et al. [43] were one of the pioneers in applying ESWT in CP. They recruited 12 children with spastic equinus foot. After a single active shock wave stimulation, a significant decrease in the MAS (from 3 to 2), an increase in the range of motion (from 20° to 50°), and an increase in the contact plantar surface area of the treated limb (from 40.3 to 80.2 cm^2^) were observed (which lasted for four weeks) in all patients. Later, Vidal et al. [44] conducted a RCT on 15 patients with spastic CP. A significant decrease in the Ashworth scale and an increase in the range of motion were observed in all patients after rESWT. They also pointed out that the positive results were maintained for two months after treatment.

Gonkova et al. [45] recruited 25 children and conducted an observational study in 2013. After rESWT, a significant increase in passive range of motion (PROM) and MAS scores were noted and remained evident until the fourth week after treatment. Baropodometric measurements also showed a significant increase in the contact plantar surface area of the affected foot and in heel pressure.

Wang et al. [46] recruited 66 patients with CP in a case control study. After treatment, patients receiving rESWT showed reduced mean MAS scores in the spastic plantar flexor muscles and increased mean PROM in their ankles. However, from a functional perspective, they found that rESWT was not superior to traditional therapy alone for improving the mean gross motor function measure (GMFM) scores of very young patients with CP.

Lin et al. [47] further addressed the effects of rESWT on CP patients who received surgical intervention for spasticity. Eighty-two children with spastic CP were recruited six weeks after multistage surgery. After ESWT, significant improvements were observed in MAS scores, GMFM scores, plantar area, and plantar pressure.

Park et al. [48] studied the effects of ESWT in CP patients according to treatment sessions. They recruited 12 patients and randomly allocated them to one- or three-session groups. Immediately after treatment and at four-week follow-up, significantly improvements in ankle PROM, mean ankle MAS, and muscle sonoelastography were noted in the three-session group, but not in the one-session group. They concluded that the therapeutic effects of ESWT on spastic CP patients were dependent on the number of ESWT sessions.

### 6.4. Spasticity in Multiple Sclerosis

Another disease that commonly causes spasticity is MS; however, research in this field is relatively scarce. Marinelli et al. [50] conducted a randomized controlled study, recruiting 68 patients with MS. Patients were divided into rESWT and placebo groups, and they were assessed at baseline, 1 week after the first session, and 1 and 4 weeks after the last session (using MAS, VAS, and H-reflex). In the rESWT group, the patients received a total of four sessions (once per week). During each session, 2000 shots were applied to the ankle extensor muscles, as well as the Achilles tendon. The frequency was 4 Hz and the pressure was 1.5 bars. While decreases in MAS and VAS were noted one week after ESWT, spinal excitability remained unaffected.

### 6.5. Botulinum Toxin Injections and EWST

Botulinum toxin (BTX) injection is a popular and effective pharmacological method of treating spasticity [49]. The reduction of spasticity is mainly caused by inhibiting acetylcholine release at the neuromuscular junction [51]. Additional distal actions with central effects were also noted in some studies, resulting in the plastic reorganization of the central nervous system [52]. Currently, BTX is expensive and not accessible in some countries [49]. As ESWT emerged as a new non-pharmacological alternative for managing spasticity [9,10], studies comparing the effects of BTX and ESWT started to appear in the literature. Of note, some authors also reported synergistic effects when the two methods were applied concomitantly.

#### 6.5.1. ESWT vs. BTX

In a randomized non-inferiority trial enrolling 42 patients with chronic stroke, Wu et al. [37] compared the effects of rESWT and BTX (Table 1 and Table 2). Their results showed that rESWT is a non-inferior treatment alternative to BTX for post-stroke upper limb spasticity. In particular, the two methods caused a similar reduction in the spasticity of wrist and elbow flexors. Furthermore, rESWT yielded a more significant improvement in the upper limb FMA score and the ROM of the wrist and elbow.

In CP patients, Vidal et al. [49] conducted a crossover study comparing the therapeutic effects of BTX and ESWT on spasticity. A total of 68 patients were initially randomly allocated to either group, while all patients also received the other treatment after six months (Table 5 and Table 6). At the end of the study, both groups had significant improvements in muscle tone and ROM over time. The authors concluded that BTX injection is not superior to rESWT for the treatment of plantar flexor muscle spasticity in CP patients.

#### 6.5.2. ESWT and BTX

A variety of adjunct therapies (electrical stimulation (ES) being the most common) following BTX administration have been proposed [53]. Santamato et al. [34] compared the effects of fESWT and ES after BTX injections in 32 patients (Table 1 and Table 2). During follow-up, patients treated with BTX and ESWT showed more significant and continuous decreases in spasticity, as assessed by MAS, spasm frequency, and pain. They reported that ESWT may enhance the effects of BTX by modulating the rheology of the muscle and the neurotransmission at the neuromuscular junction.

## 7. Limitation

There are some limitations in this review that are worth mentioning. First, we found that not all the ESWT studies on spasticity were well-constructed. Different study designs were applied in different etiologies of spasticity. For example, in post-stroke spasticity, which is the most well-studied category, we came across many well-designed randomized controlled studies. However, in papers studying patients with cerebral palsy, only three RCTs were found. Most of the other studies were placebo-controlled (2), observational (1), and case-controlled studies (1). As for multiple sclerosis, only one study, a RCT, was found. Therefore, current evidence is inconsistent, in terms of the methodology used across studies.

Second, while ESWT has effects on spasticity parameters such as MAS and MTS scores, in terms of stastical analysis, whether such parameter improvement is clinically relevant remains to be investigated. In other words, minimally clinically important differences and/or minimal detectable changes should be taken into consideration while interpreting the results. Furthermore, regarding motor control and functional recovery, mixed results were shown. The improvement in functional status from reducing spasticity may be difficult to monitor, given that many additional factors, such as compensation, may also interfere with the results. Taken together, the clinical relevance of ESWT for patients with spasticity remains uncertain.

## 8. Summary

This review shows that ESWT is a safe and effective alternative for treating spasticity caused by stroke, CP, and other UMN lesions. ESWT has prominent/direct effects on spasticity parameters such as MAS and MTS scores; however, mixed results were shown regarding functional recovery. Until now, no established practical guidelines on standard parameters exist for using ESWT in treating spasticity in different patient groups and different muscle parts. Accordingly, further comprehensive and large-scale studies are needed.

## Figures and Tables

**Table 1:** Upper limb studies: design and outcome parameters.

Author, Year, Country	Design	Grouping	Follow Up	Parameter	Safety
Santamato 2013, Italy [34]	RCT	BTX with ES (*n* = 16) BTX with fESWT (*n* = 16)	15, 30 and 90 days	MAS (+), SFS (+) VAS (+)	No adverse effect
Dymarek 2016, Poland [35]	RCT	Active rESWT (*n* = 30) Placebo rESWT (*n* = 30)	Immediately, 1 h, 24 h	MAS (+), sEMG (+) IRT (+)	No adverse effect
Li 2016, Taiwan [36]	RCT	Three rESWT sessions (*n* = 20) Single rESWT session (*n* = 20) Sham rESWT (*n* = 20)	Immediately, 1 wk, 4 wks, 8 wks, 12 wks, 16 wks	MAS (+) FMA (+)	NM
Yoon 2016, South Korea [26]	RCT	Control group (*n* = 26) Belly group (*n* = 26) Junction group (*n* = 28)	1 wk after each session(total of four evaluations)	MAS (+) MTS (+)	NM
Wu 2018, Taiwan [37]	RCT	ESWT (*n* = 21) BTX (*n* = 21)	1 wk, 4 wks, 8 wks	MAS (+), MTS (+) PROM (+), FMA (+)	No adverse effect
Park 2018, South Korea [38]	RCT	ESWT (*n* = 15) Sham-ESWT (*n* = 15)	NM	MyotonPRO (+)	NM
Li 2020, China [27]	RCT	Control (*n* = 25) Agonist (*n* = 27) Antagonist (*n* = 30)	24 h, 4 wks	MAS (+); MTS (+) VAS (+), FMA (−) Swelling scale (−)	NM
Leng 2021, China [21]	RCT	ESWT (*n* = 14) Control (*n* = 13)	Immediately, 1 wk	NeuroFlexor (+) Myotonometer (+) Electrical impedance myography (+) MAS (+), FMA (−)	NM

Abbreviations: +: statistically significant, −: statistically not significant; h: hour, wk: week; RCT: randomized controlled trial; BTX: botulinum toxin; ES: electrical stimulation; ESWT: extracorporeal shock therapy; rESWT: radial extracorporeal shock therapy; fESWT: focused extracorporeal shock therapy; MAS: modified Ashworth scale; SFS: spasm frequency scale; VAS: visual analogue scale; sEMG: surface electromyography; IRT: infrared thermal imaging; FMA: Fugl-Meyer assessment; PROM: passive range of motion; NM: not mentioned.

**Table 2:** Upper limb studies: ESWT procedures.

Author	Type of ESWT	Site of Treatment	Number, Interval of Sessions	Pulses (N)	Frequency (Hz)	Pressure (Bars)	EFD (mJ/mm^2^)
Santamato [34]	Focused	FDS	5, once every day	2000	4	1.5	0.1
Dymarek [35]	Radial	FCR, FCU, interosseous muscles	1	FCU/FCR: 1000 Intrinsic muscles: 3200	NM	1.5	0.03
Li [36]	Radial	FCR, FCU, intrinsic muscles	Group A: 3 sessions, once every week Group B: 1 session	FCR, FCU: 1500 Intrinsic muscles: 4000	5	3–3.5	NM
Yoon [26]	Radial	Elbow flexor, biceps, brachialis	3, once every week	1500	5	NM	0.068–0.093
Wu [37]	NM	FCR, FCU, biceps	3, once every week	3000 pulses (1000 on each muscle)	5	3.5	NM
Park [38]	NM	Forearm flexors, interosseous muscles	16, two times a week, total of eight weeks	Forearm flexors: 1500 Interosseous muscles: 3200 (800 each)	NM	NM	0.03
Li [27]	Radial	Agonist: biceps, brachialis, Pronator teres Antagonist: triceps	5, 4-day intervals	6000	18	1.2–1.4	0.06–0.07
Leng [21]	Radial	FCR	1	1500	4	1.5	0.038

Abbreviations: FDS: flexor digitorum superficialis; FCR: flexor carpi radialis, FCU: flexor carpi ulnaris, EFD: energy flux density; NM: not mentioned.

**Table 3:** Lower limb studies-design and outcome parameters.

Author, Year, Country	Design	Grouping	Follow-Up	Parameter	Safety
Yoon 2016, Korea [26]	RCT	Control (*n* = 26) Belly (*n* = 26) Junction (28)	1 wk after each session (total of 4 evaluations)	MAS (+) MTS (+)	NM
Taheri 2017, Iran [39]	RCT	ESWT (*n* = 13) Control (*n* = 12)	1 wk, 3 wks, 12 wks	MAS (+); VAS (+); PROM (+) 3-m walk duration (+); LEFS (+) Clonus score (−)	NM
Wu 2018, Taiwan [25]	RCT	fESWT (*n* = 15) rESWT (*n* = 16)	1 wk, 4 wks, 8 wks	MAS (+); Tardieu Scale (+) Ankle PROM (+) Dynamic foot contact area (+) 10-m walk test (−)	No adverse effect
Lee 2018, Korea [40]	RCT	ESWT (*n* = 9) Control (*n* = 9)	30 min, 1 wk, 4 wks	MAS (+); PROM (−) FMA (+) US measures (+)	NM
Radinmehr 2019, Iran [41]	RCT	US (*n* = 16) rESWT (*n* = 16)	Immediately, 1 h	H-reflex tests (−) MAS (+); PPFT (+) AROM (+); PROM (+) TUG (clinically not significant)	NM
Aslan 2021, Turkey [42]	RCT	rESWT (17) Sham (17) Control (17)	Immediately, 4 wks	MAS (+) Tardieu Scale (+) Elastography (+)	Mild pain (2)

Abbreviations: +: statistically significant, −: statistically not significant; min: minute; h: hour; wk: week; RCT: randomized controlled trial; ESWT: extracorporeal shock therapy; rESWT: radial extracorporeal shock therapy; fESWT: focused extracorporeal shock therapy; US: ultrasound; MAS: modified Ashworth scale; PROM: passive range of motion; AROM: active range of motion; FMA: Fugl-Myer assessment; PPFT: passive plantar flexor torque; LEFS: lower extremities functional scale; PPFT: passive plantar flexor torque; TUG: timed “up and go” test; NM: not mentioned.

**Table 4:** Lower limb studies-ESWT procedures.

Author	Type of ESWT	Site of Treatment	Number, Interval of Sessions	Pulses (N)	Frequency (Hz)	Pressure (Bars)	EFD (mJ/mm^2^)
Yoon [26]	Radial	Knee flexors, semitendinosus muscles	3, once every week	1500	5	NM	0.068–0.093
Taheri [39]	NM	GN	3, once every week	1500	4	NM	0.1
Wu [25]	Focused Radial	GN and soleus	3, once every week	3000 (1500 per muscle)	5	rESWT: 2	fESWT: 0.10
Lee [40]	NM	GN	1	2000	4	NM	0.1
Radinmehr [41]	Radial	GN	1	2000	5	1	0.340
Aslan [42]	Radial	Ankle flexor	4, twice per week	1500	10	2	NM

Abbreviations: GN: gastrocnemius; EFD: energy flux density; NM: not mentioned.

**Table 5:** Cerebral palsy studies: design and outcome parameters.

Author, Year, Country	Design	Subjects/Grouping	Follow-up	Parameter	Safety
Amelio 2010, Italy [43]	Prospective, placebo-controlled study	*n* = 12	Immediately after placebo, immediately after ESST, 1 wk, 4 wks, 12 wks	MAS (+) PROM (+) Pedobarometric assessment (+)	NM
Vidal 2011, Spain [44]	Placebo-controlled clinical trial	*n* = 15	1 mo, 2 mos, 3 mos	MAS (+) ROM (+)	NM
Gonkova 2013, Italy [45]	Observational study	*n* = 25	2 wks, 4 wks	MAS (+), PROM (+) Baropodometric measurements (+)	NM
Wang 2016, China [46]	Case-control study	rESWT (*n* = 34) Control (*n* = 32)	1 mo, 3 mos	MAS (+) PROM (+) GMFM-88 (−)	No adverse effect
Lin 2018, China [47]	RCT	rESWT (*n* = 43) Control (*n* = 39)	2 wks, 1 mo	GMFM (+), MAS (+) Plantar area and pressure (+)	NM
Park 2018, Korea [48]	RCT (a pilot study)	1 ESWT (*n* = 6) 3 ESWT (*n* = 6)	Immediately after the first and third ESWT, 4 wks	MAS (+) PROM (+) Sonoelastography (+)	No adverse effect
Vidal 2020, Germany [49]	RCT, cross-over study *	BTX-A (*n* = 33) rESWT (*n* = 35)	3 wks, 2 mos, 3 mos	Tardieu scale, with goniometer (+)	NM

Abbreviations: +: statistically significant, −: statistically not significant mo: month; wk: week; RCT: randomized controlled trial; ESWT: extracorporeal shock therapy; rESWT: radial extracorporeal shock therapy; MAS: modified Ashworth scale; PROM: passive range of motion; GMFM-88: gross motor function measure-88, NM: not mentioned. * Crossover six months later.

**Table 6:** Cerebral palsy studies-ESWT procedures.

Author	Type of ESWT	Site of Treatment	Number, Interval of Sessions	Pulses (N)	Frequency (Hz)	Pressure (Bars)	EFD (mJ/mm^2^)
Amelio [43]	NM	GN and soleus	1, one placebo session, followed 6 wks later by one active session	1500 per muscle	NM	NM	0.030
Vidal [44]	Radial	Biceps brachii, wrist flexors, hip adductors, GN, soleus, and hamstrings	3, once every week	2000 per muscle	8	2	0.10
Gonkova [45]	Radial	GN and soleus muscle	1, one placebo session, 1 active session 4 wks later	1500 per muscle	5	1.5	NM
Wang [46]	Radial	plantar flexor, GN	12, one ESWT session per week for 3 months,	1500 permuscle	8	0.6	0.03
Lin [47]	Radial	Hamstring	4, once a week	2000	10	2	NM
Park [48]	NM	GN	1 or 3 sessions, once a week	1500	4	NM	0.030
Vidal [49]	Radial	GN and soleus muscle	3, one session per week	2000	8	2.2–2.4	0.10–0.12

Abbreviations: GN: gastrocnemius; EFD: energy flux density; wks: weeks; NM: not mentioned.
